# Prevalence of Hepatitis B Virus and Hepatitis C Virus in a Tea Garden of Bangladesh

**DOI:** 10.5005/jp-journals-10018-1227

**Published:** 2017-05-05

**Authors:** Mamun Al Mahtab, Shamima Akhter, Kutub U Mollick, Mohammad H Uddin, Sakirul I Khan, Sheikh MF Akbar

**Affiliations:** 1 Department of Hepatology, Bangabandhu Sheikh Mujib Medical University, Dhaka, Bangladesh; 2 Department of Physiology, Dhaka Medical College, Dhaka, Bangladesh; 3 Department of Hepatology, Khulna Medical College, Khulna, Bangladesh; 4 Forum for the Study of the Liver, Dhaka, Bangladesh; 5 Department of Anatomy and Embryology, Ehime University Graduate School of Medicine Toon, Ehime Japan; 6 Department of Medical Sciences, Toshiba General Hospital, Tokyo, Japan

**Keywords:** Epidemiological prevalence, Hepatitis B virus, Hepatitis C virus, Tea garden.

## Abstract

**Introduction:**

The overall health status of workers of tea garden of Bangladesh is below the national standard. Also, almost nothing has been reported about status of hepatitis virus infection among these population and there is also a lack of consensus.

**Materials and methods:**

Several health-related facts, especially those of liver diseases, were collected from 130 workers of tea garden via questionnaire. Sera were also collected from these subjects to assess positivity of hepatitis B surface antigen (HBsAg) and antibody to hepatitis C virus (anti-HCV). Hepatitis B virus (HBV) genotype was also done using genotype-specific primers in HBsAg-positive sera.

**Results:**

Out of 130 tea garden workers, 5 were positive for HBsAg; however, none was reactive to anti-HCV. Genotyping of HBV deoxyribonucleic acid of 4 sera samples revealed that 2 belonged to genotype A, 1 to genotype C, and 1 to genotype D. Various risk factors were documented in HBV-infected subjects by analyzing the questionnaire.

**Conclusion:**

Hepatitis B virus in considerable high percentage is prevalent among workers of tea garden in Bangladesh, and immediate vaccination against HBV should be employed. Also, health education system should be accentuated in specific population like tea garden workers.

**How to cite this article:** Al Mahtab M, Akhter S, Mollick KU, Uddin MH, Khan SI, Akbar SMF. Prevalence of Hepatitis B Virus and Hepatitis C Virus in a Tea Garden of Bangladesh. Euroasian J Hepato-Gastroenterol 2017;7(1):107-110.

## INTRODUCTION

Although liver diseases are common in Bangladesh, the prevalence of hepatitis B virus (HBV) and hepatitis C virus (HCV) has shown marked variation among studies and in different parts of this country.^1-3^ Rudra et al^1^ have shown that 126 of 2,015 subjects had HBV. On the contrary, Ashraf et al^2^ reported hepatitis B surface antigen (HBsAg) positiv-ity at 0.7% individuals of Bangladesh. Mahtab et al^3^ showed that HBsAg positivity is about 5.5% among the general population in Bangladesh. Definitely, these diversities of HBV prevalence may be explained by subject selection diversity. However, there is the paucity of information about the prevalence of HBV and HCV among specific population group/groups. The tea gardens and their entities have very specific features in Bangladesh. Considerable numbers of laborers of tea gardens came from different parts of India in early- to mid-19th century. Subsequently, local people also worked in different jobs in the tea gardens. The workers, especially the laborers of tea gardens, have been discriminated in different aspects during British rule and Pakistani period. The same trends seem to be prevailing at present: The overall health situations of the laborers are bad considering the national average. This study was undertaken to develop insights about HBV and HCV status in one of the oldest tea gardens of Bangladesh and to have some idea about ways to tackle these.

## STUDY DESIGN, RESULTS, AND DISCUSSION

**Figs 1A to C:**
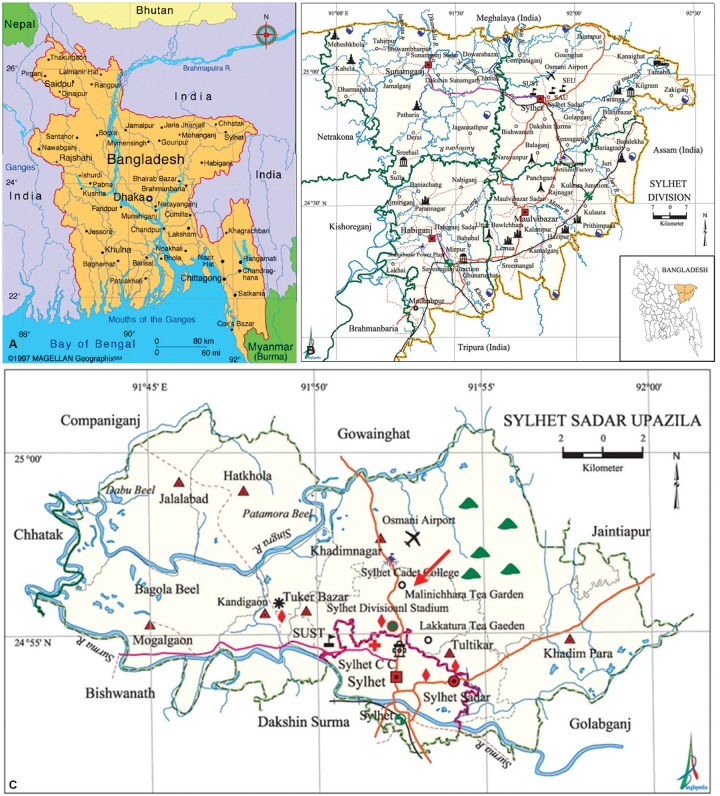
(A) Map of Bangladesh; (B) Map of Expanded Sylhet District; and (C) Map of Sylhet Sadar Upazila

The workers of Malnicherra Tea Garden, Sylhet, (Fig. 1A), were requested to respond to several questions for getting insights about liver diseases. Malnicherra is within administrative control of Sylhet Division (Fig. 1B) in broad sense and specifically, under Sylhet Sadar Upazila (Fig. 1C). Malnicherra is shown by a red arrow in Figure 1C. Sera were collected from the workers of Malnicherra tea gardens and checked for HBsAg and anti-HCV. The HBV genotype was accomplished in sera that were expressing HBsAg with detectable levels of HBV deoxyribonucleic acid (DNA).

**Fig. 2:**
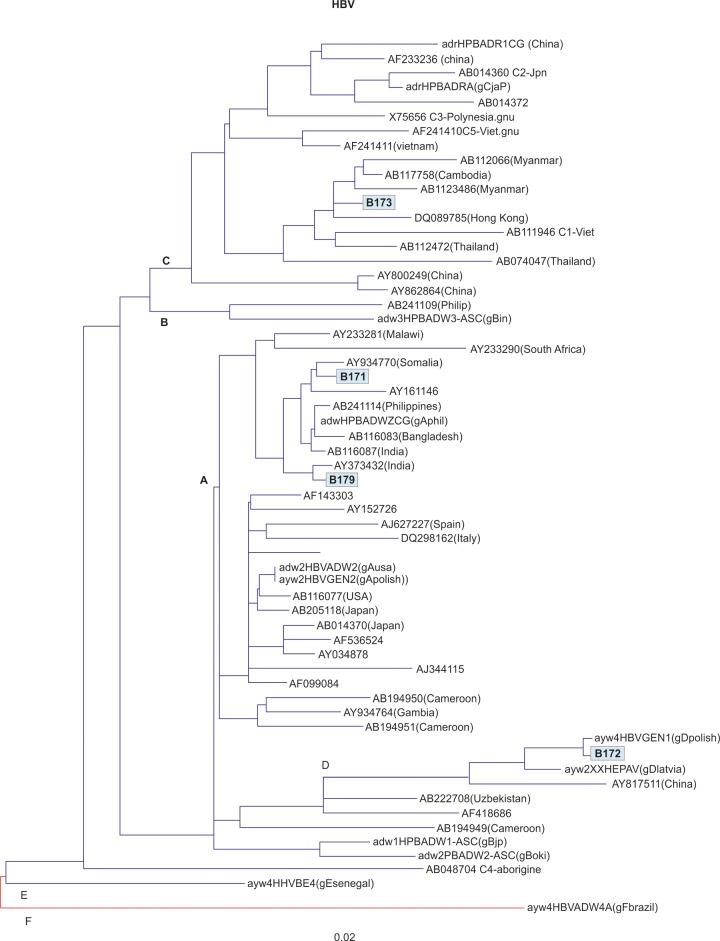
Phylogenetic tree showing genomic analysis of HBsAg-positive samples for genotyping

Out of 130 subjects, whose sera were collected, 5 were expressing HBsAg in the sera (3.85%). However, anti-HCV was not detected in any sera. The HBV DNA was isolated from the sera of 5 HBsAg-positive subjects, and HBV genotyping was done in 4 of the 5 subjects. The HBV genotypes of A, C, and D were detected in 2, 1, and 1 subjects respectively (Fig. 2).

To assess the risk factors, we used a questionnaire and it was found that 43 patients had history of jaundice and almost all patients received treatment by quack. History of infusion, surgery, vaccinations, use of barber shop for shaving, and delivery by traditional birth attendant (TBA) was predominant, as shown in Table 1. Also, analysis of 5 HBsAg-positive subjects showed prevalence of more than five risk factors; however, it remains elusive if these had any specific role in HBV transmission. Although 16 subjects had history of intravenous drug transfusion, HCV infection was not detected in any subject, indicating low prevalence of HCV in Bangladesh. However, specific factors related to these remain to be resolved. This is a preliminary study, and limited approaches have been made to assess HBV and HCV infection in specific population group of Bangladesh. Indeed, there are several such population groups and also ethnic population in Bangladesh and study about distribution of various diseases, including hepatitis viruses, is urgently warranted.

**Table 1:** **Table 1:** Risk factors of HBV and HCV infection (n = 130)

*Parameters*		*Values*	
Age		28 ± 15 years	
Sex (male:female)		63:67	
Family history of liver disease		3	
History of jaundice		43	
History of blood transfusion		5	
Treatment by quack doctor		127	
History of previous surgery		31	
History of dental procedure		20	
History of body piercing		61	
Tattooing		5	
Alcohol consumption		16	
Intravenous drug abuse		0	
Circumcision by traditional system		15	
Delivery by TBA		25	
Cholera vaccine		55	
Small pox vaccine		49	
History of injection		92	
History of infusion		39	
Shaving at barber shop		37	
